# Gain of function in somatic *TP53* mutations is associated with immune‐rich breast tumors and changes in tumor‐associated macrophages

**DOI:** 10.1002/mgg3.1001

**Published:** 2019-10-22

**Authors:** Michael Behring, Ana I. Vazquez, Xiangqin Cui, Marguerite R. Irvin, Akinyemi I. Ojesina, Sumit Agarwal, Upender Manne, Sadeep Shrestha

**Affiliations:** ^1^ Department of Epidemiology University of Alabama at Birmingham Birmingham AL USA; ^2^ Department of Pathology and Surgery University of Alabama at Birmingham Birmingham AL USA; ^3^ Institute for Quantitative Health Science and Engineering Michigan State University East Lansing MI USA; ^4^ Department of Epidemiology and Biostatistics Michigan State University East Lansing MI USA; ^5^ Department of Biostatistics and Bioinformatics Emory University Atlanta GA USA; ^6^ HudsonAlpha Institute for Biotechnology Huntsville AL USA; ^7^ Comprehensive Cancer Center University of Alabama at Birmingham Birmingham AL USA

**Keywords:** breast cancer, *TP*53 gain of function, tumor‐associated macrophages, tumor‐invasive lymphocytes

## Abstract

**Background:**

Somatic mutations in *TP53* are present in 20%–30% of all breast tumors. While there are numerous population‐based analyses of *TP53*, yet none have examined the relationship between somatic mutations in *TP53* and tumor invasive immune cells.

**Methods:**

Clinical and genetic data from 601 women drawn from The Cancer Genome Atlas (TCGA) were used to test the association between somatic *TP53* mutation and immune‐rich or immune‐poor tumor status; determined using the CIBERSORT‐based gene expression signature of 22 immune cell types. Our validation dataset, the Molecular Taxonomy of Breast Cancer International Consortium (METABRIC), used a pathologist‐determined measure of lymphocyte infiltration.

**Results:**

Within *TP53*‐mutated samples, a mutation at codon p.R175H was shown to be present at higher frequency in immune‐rich tumors. In validation analysis, any somatic mutation in *TP53* was associated with immune‐rich status, and the mutation at p.R175H had a significant association with tumor‐invasive lymphocytes. TCGA‐only analysis of invasive immune cell type identified an increase in M0 macrophages associated with p.R175H.

**Conclusions:**

These findings suggest that *TP53* somatic mutations, particularly at codon p.R175H, are enriched in tumors with infiltrating immune cells. Our results confirm recent research showing inflammation‐related gain of function in specific *TP53* mutations.

## Graphical abstract

The occurrence of somatic mutations in *TP53* was found to be higher in immune‐rich breast tumors. We found that a single somatic variant of *TP53*, p.R175H, occurs at a higher frequency in immune‐rich breast tumors. This mutation was also associated with a decreased proportion of tumor‐invasive M1 macrophages, and increased M0 macrophages.

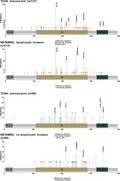

## 1 INTRODUCTION

*TP53* (OMIM:191170) encoding tumor protein p53 functions primarily as a transcription factor to regulate cellular homeostasis. Somatic mutations in *TP53* are frequent in breast cancer. According to the type of stress as well as cellular environment, the function of *TP53* can vary from cell death, DNA repair, halting or terminating cell cycle, and maintenance of metabolic equilibrium (Muller & Vousden, 2014; Robles & Harris, 2009). The effects of somatic alterations in *TP53* have been linked to all stages along the oncogenic timeline of breast tumors; from early tumorigenesis, through growth and development, to distant metastasis (Walerych, Napoli, Collavin, & Del Sal, 2012). In population‐based research, molecular subtypes of breast cancer were shown to have *TP53* mutants differing in frequency, type, and function (Dumay et al., 2013). Specific mutations in the L2 (codons 164–194) and L3 motifs (codons 237–250) in *TP53* are independently associated with poor survival (Olivier et al., 2006). Gain of function (GOF) in specific *TP53* mutations, observable through alterations in the inflammatory response, can alter further mutation through inflammation‐activated factors in a cyclical manner (Rahnamoun et al., 2017). In breast cancer, mutated *TP53* can cause loss of ability to regulate TNF signaling, followed by NF‐kB activation and a perpetuated inflammatory cycle, linked to recruitment of immune cells within the tumor (Di Minin et al., 2014).

Along with the identification of individual genomic markers of immune‐rich tumors, total mutational burden as well as clonal populations have been shown as important factors in immune response. Both characteristics are measures of tumor heterogeneity. While somatic mutations are a marker of genetic damage, it is this very damage that can result in the creation of tumor‐specific neoantigens, which facilitate recognition by the immune system (Schumacher & Schreiber, 2015). The greater the somatic mutational burden, the more neoantigens are created, and the more likely a T‐cell immune response is to occur. Previous research has shown a link between somatic mutation, neoantigens, and immune cell infiltration in multiple types of tumors, including those of the breast (Brown et al., 2014; Heemskerk, Kvistborg, & Schumacher, 2013). The clonal consistency of mutations across the cells of a tumor is another measure of heterogeneity which affects immunogenicity. Measures of clonal heterogeneity vary from tumor‐wide, to the estimates of single nucleotide variant in each cancer cell (Mroz & Rocco, 2013). In breast cancer, lower overall clonal heterogeneity has been associated with immune‐rich status across all subtypes (Karn et al., 2017a; Safonov et al., 2017).

The aim of this analysis was to investigate the association between *TP53* somatic mutations and immune cell infiltration in breast tumors. We used an established immune gene expression signature to characterize all tumors as immune‐rich or immune‐poor, and compared differences across the spectrum of *TP53* somatic mutations. An important part of this analysis was the validation of our results in another set of data which used pathologist‐derived measures of tumor lymphocytic invasion.

## 2 METHODS

Clinical and genetic data from 601 women with invasive ductal carcinoma and no history of other malignancy or therapy were drawn from The Cancer Genome Atlas (TCGA) (TCGA Research Network: http://cancergenome.nih.gov). Molecular subtype of the tumors was based on immunohistochemistry (IHC) measurements. Estrogen receptor‐positive (ER^+^) or progesterone receptor‐positive (PR^+^) and Her2 ± were grouped together in a luminal category. Women with negative ER, PR, and Her2 IHC were considered triple‐negative (TNBC) and women who were Her2 + only were considered Her2 type. Tumor stage followed the AJCC 7th edition guideline and was recorded as reported from the participating institutions for TCGA. Lymph node metastasis was used as a yes/no variable based on TCGA clinical data and AJCC staging TNM (tumor, lymph node, metastasis) criteria (Edge & Compton, 2010).

RNA‐Seq data and *TP53* sequence data were obtained from the GDC Data Portal (https://portal.gdc.cancer.gov). Using the MuSE pipeline from Baylor College (Fan et al., 2016), somatic variants were identified by comparing normal to tumor sequence data, in binary alignment map (BAM) files for each sample. Somatic DNA sequence variations were compared against GenBank reference sequence NM_000546.5, and classified using the Human Genome Variation Society (HGVS) short nomenclature (http://www.hgvs.org/mutnomen/recs-DNA.html). TCGA Level 3 RNAseqV2 expression data files (collected using Illumina HiSeq 2000 platform) provided normalized mRNA expression counts. CIBERSORT, a machine‐learning method that estimates the proportion of specific cell types using mRNA data, has been shown to be a reliable marker for tumor immune cell deconvolution across multiple cancer types (Gentles et al., 2015). The LM22 reference profile (based on patterns from 527 signature genes in 22 immune cell types) verified by Gentles et al.(Gentles et al., 2015) was used to define immune cell infiltrating and noninfiltrating tumors. Similarity of the real gene expression of 527 signature genes in a patient was compared to the reference RNA‐signature profile of 22 immune cell types and was then compared to randomly chosen genes tested 1,000 times. For each tumor, the real genes were then tested against a null distribution of permutations to define immune‐rich and immune‐poor status using a significance threshold of 0.05. In addition, the calculated proportion of immune cell type per sample was used to represent the composition of immune cells in immune‐rich tumors.

Total mutations per tumor sample were calculated from this data with the inclusion of silent mutations. This was a measure of genome‐wide somatic mutation in an effort to indicate mutational burden. While the consequence of synonymous/silent mutations upon immune response is unclear (Gotea, Gartner, Qutob, Elnitski, & Samuels, 2015; Rooney, Shukla, Wu, Getz, & Hacohen, 2014), they were included to account for any indirect connection to the mutational process in each patient (Chalmers et al., 2017). Since overall, genome‐wide, clonal heterogeneity has been shown as different by molecular subtype (Karn et al., 2017b; Safonov et al., 2017), the estimated variant allele proportion of total reads for *TP53* was used as a measure of clonality of *TP53* mutations throughout the tumor sample.

A second set of data from the Molecular Taxonomy of Breast Cancer International Consortium (METABRIC) (Curtis et al., 2012) was used to validate our initial findings. In a subset of 1,105 METABRIC samples of frozen tissue sections, hematoxylin and eosin (H&E) staining was performed to determine the level lymphocytic infiltration, as previously described (Silwal‐Pandit et al., 2014). In order to better match our TCGA data variable of immune‐rich/immune‐poor, the original categories of absent, mild, and severe lymphocytic infiltration were combined to a dichotomous measure of absent or present for this study. In TCGA data, immune‐rich indicates tumors with an RNA expression signature significantly matching that of 22 leukocyte immune cell type signature matrix. In METABRIC, tumors were evaluated specifically for lymphocytes, morphologically, by pathologists. *TP*53 nucleic acid sequence was manually curated by previous authors for the coding region from exons 2–11 (Silwal‐Pandit et al., 2014). In this study, molecular subtypes were based on IHC and expression of ER, PR, and Her2 receptors to create similar categories as TCGA; Her2, Luminal, and TNBC. Expression‐based (PAM50) categories of molecular subtype signatures were also available from METABRIC data (Silwal‐Pandit et al., 2014), and were used in qualitative review of results.

In both sets of data, the connection between somatic, immune, and clinical variables across immune signature/ lymphocytic infiltration status was assessed using chi square tests and Fisher's exact test for categorical variables, and *F*‐tests for all continuous measures. In subanalysis of only *TP53*‐mutated samples, each individual codon with mutational count over 5 was chosen, and then tested against all other *TP53* codons using a Fisher's exact test. METABRIC data were evaluated using the same analysis in order to validate initial TCGA results. The covariate‐adjusted association of *TP53* mutation with tumor immune invasion was tested in both sets of data using a logistic regression model with immune status as a binary predictor variable. Interaction terms for *TP53* mutation status and receptor subtype were considered in both sets of data. In TCGA data only, total genome‐wide counts of mutations per tumor (mutational burden), with the inclusion of silent mutations, were tested in a generalized linear model using Poisson regression. Wilcoxon rank sum test was used to measure differences in immune status groups by variant allele proportion of total reads. A subanalysis in TCGA immune‐rich tumors was done to compare proportion of CIBERSORT‐determined immune cell type across several groups: any mutation in *TP53* versus *TP53* wild type, and versus individual *TP53* codons. This was done using Kruskal–Wallis tests in pairwise analysis. Pearson correlation tests between cell types in these groups were also performed. Using TCGA‐provided IHC measures of percent cellularity; we evaluated the relationship between CIBERSORT Pearson correlation coefficient (indicating immune‐rich, and immune‐poor signature) and IHC tumor cellularity with the Wilcoxon rank sum. In METABRIC, categorical tumor cellularity was compared against tumor lymphocytic invasion using a chi square test.

## 3 RESULTS

**Figure 1:**
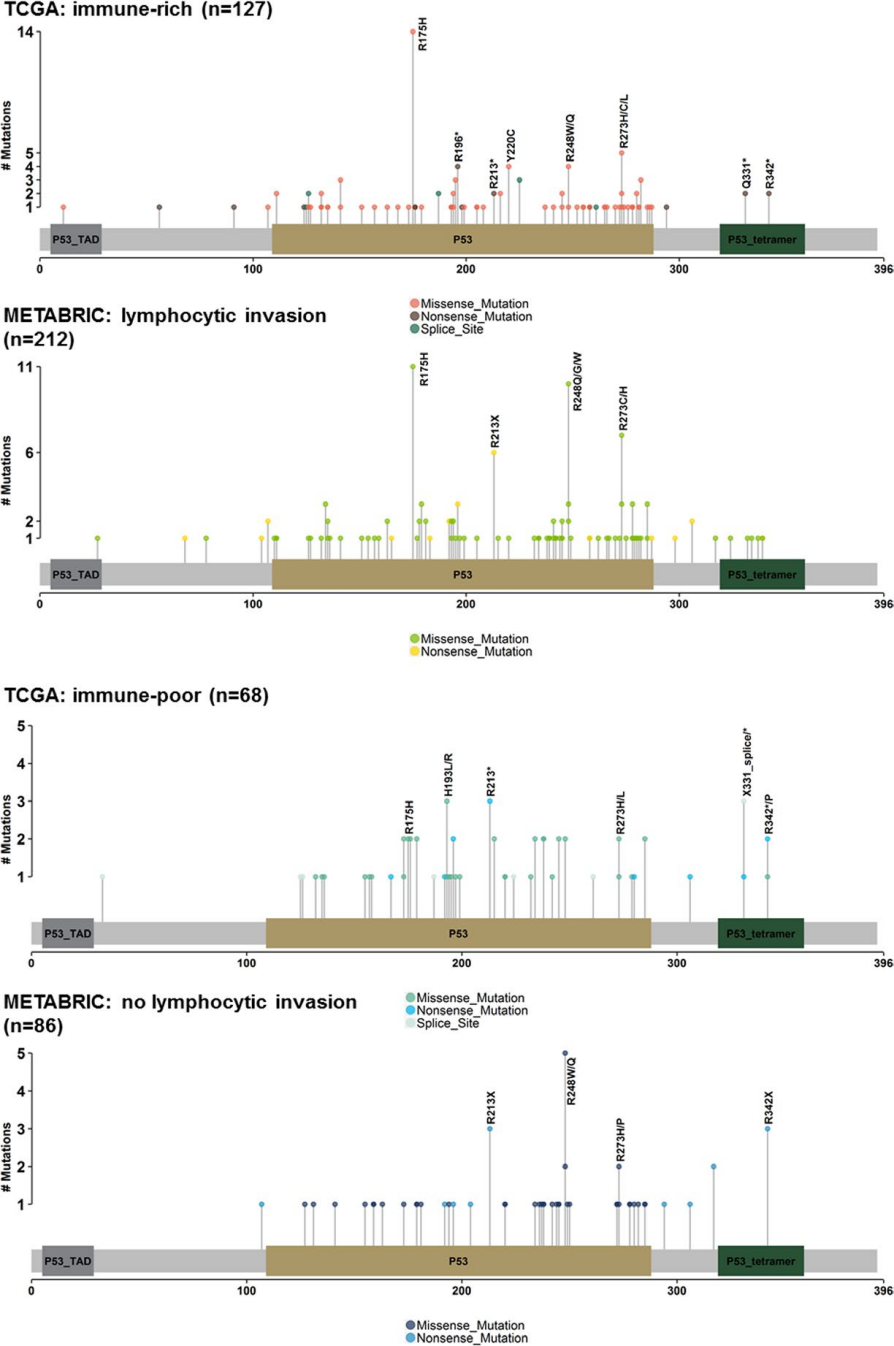
Frequency of mutations in *TP53* by codon in tumors by immune signature status in The Cancer Genome Atlas (TCGA) or determined to have lymphocytic invasion in The Molecular Taxonomy of Breast Cancer International Consortium (METABRIC) data

The average age of the participants was 56 years, with approximately 75% of women being White, early (I or II) stage, or Luminal. Of the 601 TCGA participants, 299 had the tumors characterized as immune‐rich. Occurrence of at least one somatic mutation in the *TP53* was noted in 32% of patients. Median count of genome‐wide somatic mutation (mutational burden) per bulk tumor was 29 mutations. Women with any mutation in *TP53* had 2.37 times higher odds of also having immune‐rich tumors (95% CI 1.76–3.68, *p* < .001). Consistent with previous research findings, immune‐rich tumors had a higher total relative mutational burden and lower clonality as measured by variant allele percent of total reads (*p* < .001 for Poisson and Wilcoxon, respectively) (Table 1). Patient age at diagnosis and race had no influence on immune signature status. Logistic regression models adjusted for age, and pathologic stage showed that any *TP53* mutation was independently associated with odds of tumors having immune‐rich signature (2.64, 95% CI 1.84–3.81, *p* < .001, Table 3). Interaction terms for *TP53* mutational status and receptor subtype were all found to be nonsignificant. In *TP53* mutation codon‐level analysis, the most common *TP53* somatic mutant was p.R175H, found in 8% (16/195) of all women. The majority of p.R175H somatic mutants (14/16) was found in immune‐rich tumors (Fisher's *p* value .058, Table 3 and Figure 1). Furthermore, most (63%) were from the combined Luminal (A and B) subtypes (not shown). Compared against all other *TP53* mutations, in Luminal type tumors, p.R175H had a significant association with immune‐rich status (Fisher's *p*‐value .041).

**Table 1:** Characteristics of study participants in The Cancer Genome Atlas (TCGA) and The Molecular Taxonomy of Breast Cancer International Consortium (METABRIC)

	TCGA			METABRIC		
Clinical and molecular variables	Immune‐rich (*n* = 299)	Immune‐poor (*n* = 302)	*p*‐value	lymphocytic invasion present (*n* = 713)	lymphocytic invasion absent (*n* = 392)	*p*‐value
Age at diagnosis (mean, IQR)	56 (47, 65)	57 (47, 65)	.781	60 (50, 72)	62 (53, 71)	.192
*TP53* somatic mutation (any codon)						
Yes	127 (43)	68 (22)	<.001	212 (30)	86 (22)	.006
Lymph node metastasis						
Yes	152 (51)	183 (61)	.016	299 (56)	122 (40)	<.001
Race^a^						
Asian	22 (8)	23 (8)	.696	8 (2)	12 (5)	.206
African American/ African European	40 (15)	34 (12)		1 (0.3)	0 (0)	
Caucasian/European	210 (77)	221 (80)		355 (95)	205 (91)	
European/Asian	–	–		3 (0.8)	2 (0.9)	
Receptor subtype^b^						
Her2	18 (7)	9 (3)	<.001	43 (8)	18 (6)	<.001
Luminal	198 (74)	257 (90)		382 (71)	255 (84)	
TNBC	51 (19)	20 (7)		112 (21)	30 (10)	
Total mutations per bulk tumor (median, IQR)	36 (21, 66)	26 (18, 42)	<.001			
*TP53* variant allele percent (median, IQR)^c^	44 (33, 55)	61 (51, 76)	<.001			

a Approximately 9% of race for TCGA and ~ 50% for METABRIC were missing; however, the relationship of missing race across both *TP53* and immune status was determined to be random for TCGA.

b In METABRIC, approximately 25% of receptor subtype data were missing in both groups of lymphocytic invasion.

c For *TP53* mutant‐only analysis (*n* = 195).

In validation analysis using METABRIC data, 27% of all patients (298/1105) had at least one somatic mutation in *TP53*. Women with *TP53* mutation had 1.51 times the odds of also having tumors with lymphocytic infiltration (95% CI 1.42–1.89, *p*‐value .005). Adjusted models in METABRIC validated our original TCGA findings that women with any mutation in *TP53* had higher odds of lymphocytic invasion in tumors (1.43, 95% CI 1.01–2.03, *p*‐value .044, Table 3), see Table 2. No significant effect modification was found between *TP53* mutation status and receptor subtype. In codon‐specific analysis of women with *TP53* mutations only, the most frequently mutated codon locations in *TP53* were at p.R248Q (12/298) and p.R175H (11/298) (Fisher's *p*‐values of .022 and .032, respectively). All of the mutations at codon p.R175H were also in tumors with lymphocytic invasion (11/11), see Table 3. Interestingly, in a subset of only women with *TP53* somatic mutations, both Luminal B (4/11) and Basal (5/11) subtypes exhibited codon p.R175H mutations most often.

**Table 2:** Adjusted odds of tumor immune infiltration for TCGA and METABRIC

TCGA data	Odds of immune‐rich tumor signature	95% LL	95% UL	*p*‐value
*TP53* ( any somatic mutation vs. wild type)	2.64	1.84	3.81	1.47E−07
Age at diagnosis (per year increase)	1.00	0.99	1.01	.852
Tumor stage (III & IV vs. I& II)	0.56	0.37	0.84	.006

METABRIC data	Odds of tumor lymphocytic invasion	95% LL	95% UL	*p*‐value
*TP53* ( any somatic mutation vs. wild type)	1.43	1.01	2.03	.044
Age at diagnosis (per year increase)	1.00	0.98	1.01	.392
Tumor size (per mm increase)	0.99	0.98	1.00	.039
Lymph node metastasis ( any vs. none)	2.01	1.48	2.73	.00000856
Grade (one unit increase 1–3)	1.31	1.00	1.72	.048

**Table 3:** Most frequently occurring mutations (≥5) by codon by tumor immune infiltration status, in *TP53* mutation‐only analysis of TCGA and METABRIC data

Codon	TCGA (*n* = 195)				METABRIC (*n* = 298)			
	Tumor immune signature				Lymphocytic invasion			
	Immune‐poor (*n* = 68)	Immune‐rich (*n* = 127)	Total	Fisher *p*‐value	Absent (*n* = 86)	Present (*n* = 212)	Total	Fisher *p*‐value
p.R248Q	2	1	3		2	10	12	
p.R175H	2	14	16	.058	0	11	11	.037
p.R213X	3	2	5		3	6	9	
p.R248W	0	4	4		5	2	7	.022
p.R273C	0	2	2		0	7	7	
p.R273H	2	5	7		2	3	5	
p.R196X	2	4	6		1	3	4	
p.Y220C	1	4	5		1	2	3	

## 4 DISCUSSION

This study is one of the first to evaluate the relationship between the codon‐level *TP53* somatic mutations and RNA‐signature/pathologist‐confirmed markers of tumor immune cell infiltration on a population level. We observed that somatic *TP53* mutation in both cohorts of women with breast cancer was consistently associated with tumors having immune cell infiltration, and this relationship was not modified by receptor subtype. The p.R175H mutation in *TP53* could be involved in GOF through the immune response or transcription mechanism as the mutation is located in L2 motif.

Part of the evidence for *TP53* somatic mutations and immune modulation in breast cancer comes from research using p.R175H cell lines (Di Minin et al., 2014; Lu, Liu, & Xu, 2013; Weisz et al., 2007). Our validated results suggest that the tumor immune infiltrating status of codon p.R175H of *TP53* may be a marker of GOF and the chronic immune signaling cycle. Previous research using the same dataset has shown wild‐type *TP53* and basal‐like breast tumors to be associated with cytotoxic T‐cell signatures, and that basal‐like tumors had a high frequency of p.R175H mutations (Lu et al., 2013). While we observed no overall effect of receptor subtype on the association between *TP53* mutations and tumor immune status, codon‐specific analysis showed that p.R175H was most frequent in luminal and basal subtypes.

**Figure 2:**
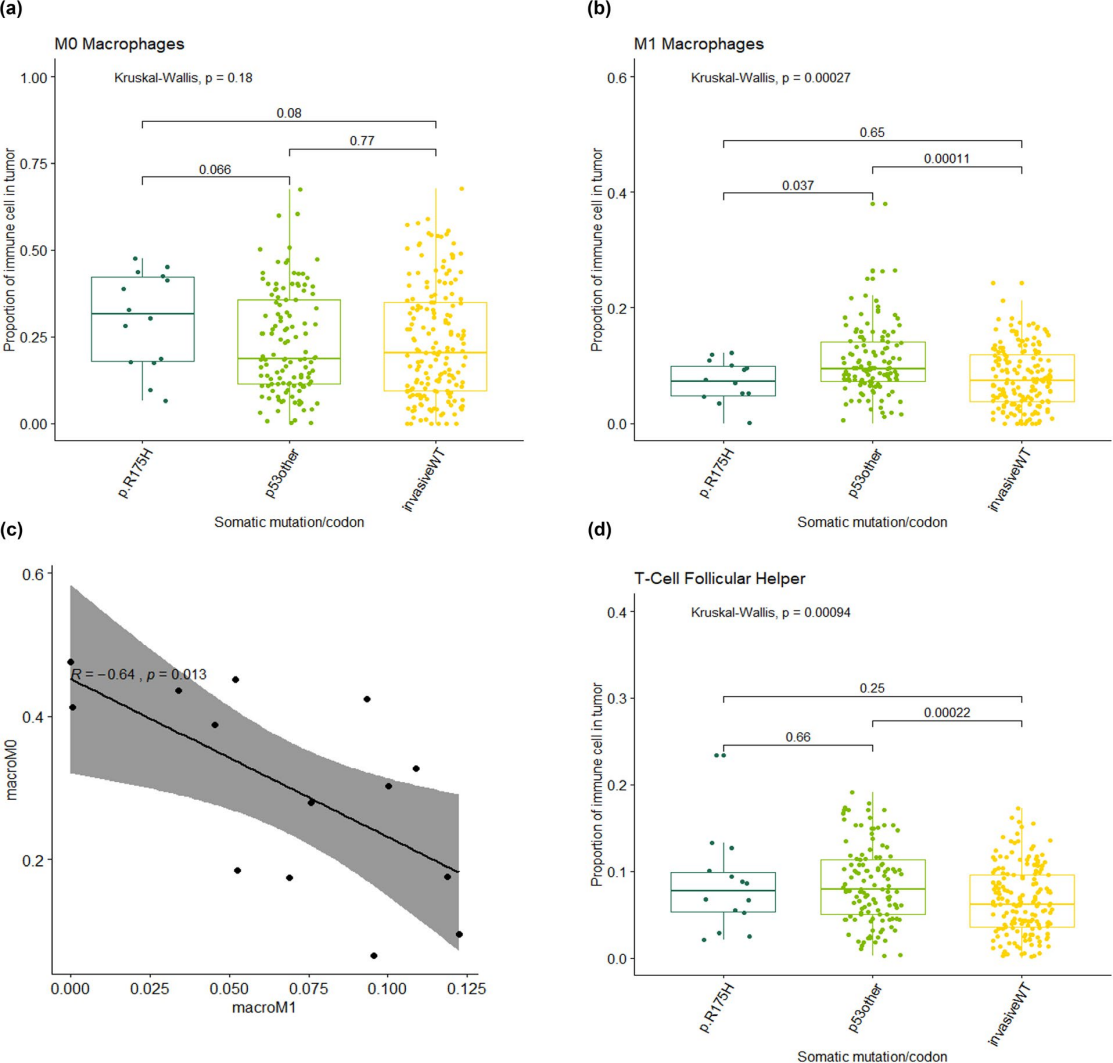
Proportion of immune cell type in immune‐rich tumors by wild‐type *TP53* (invasive WT), codon 175 (p.R175H) and somatic mutations in all other *TP53* codons (p53other). (a) Proportion of M0 macrophage; (b) proportion of M1 macrophages; (c) Correlation between M0 and M1 macrophage proportions in p.R175H samples, *n* = 14; (d) Proportion of follicular helper T cells. For (a), (b), (d) statistical tests *p*‐values are shown for pairwise and overall

**Figure 3:**
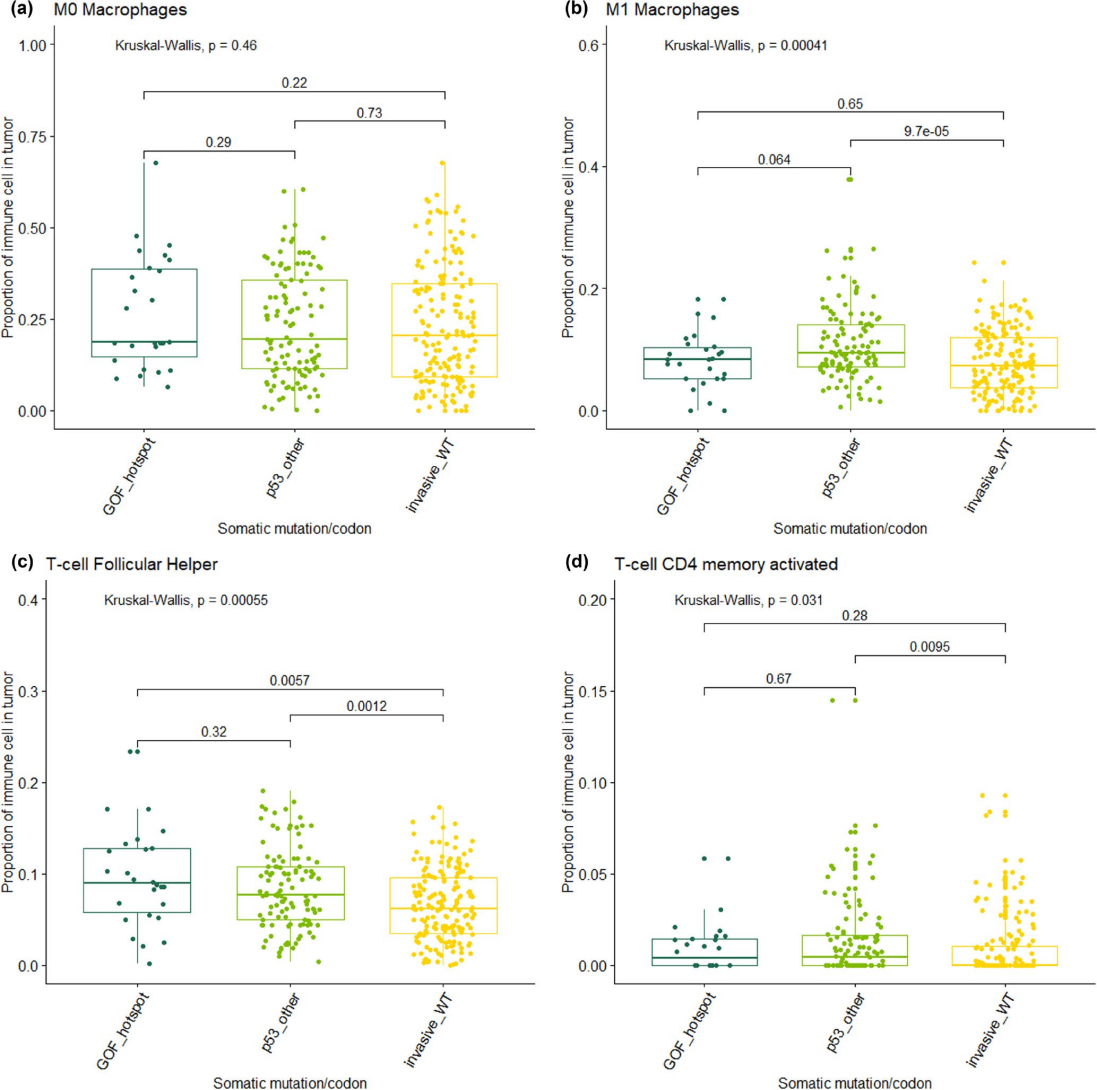
Proportion of immune cell type in immune‐rich tumors by wild‐type *TP53* (invasive_WT), Gain of Function hotspot mutations (GOF_hotspot) and somatic mutations in all other *TP53* codons (p53_other). (a) Proportion of M0 macrophage; (b) proportion of M1 macrophages; (c) Proportion of follicular helper T cells; (d) proportion CD4 memory activated T cells. For (a)‐(d) statistical test *p*‐values are shown for pairwise and overall

Our findings of increased M0 (not activated) and decreased classically activated M1 macrophages in tumors with p.R175H mutations are consistent with previous literature. Studies have shown not only an increase of M0 and M2 macrophages in *TP53*‐mutated tumors, but also a reprogramming of these cells associated with decreased phagocytic capacity, enhanced ECM degradation, higher invasiveness, and worse survival (Cooks et al., 2018). When compared to all other *TP53* mutations and wild‐type tumors, p.R175H tumors had a higher proportion of inactive M0 macrophages (*p*‐value .06), as well as decreased activated M1 macrophages (*p*‐value .03). The proportion of M0 macrophages was found to be inversely correlated with the presence of M1 macrophages in patients with mutations at p.R175H. Follicular helper T cells were found in a higher proportion in all other *TP53* somatic mutations compared to *TP53* wild‐type tumors (Figure 2). Additional analysis comparing all other *TP53* mutations, wild‐type tumors and *TP53* hotspot mutations (p.R175H, p.R248Q, p.R273H, p.R273C, and p.R282W) found that M0 and M1 immune cell proportions did not differ across the hotspot groups versus others, suggesting that the relationship between M0 and M1 macrophages is unique to p.R175H (Figure 3).

We were limited by the insufficient size of the Her2 group for our analysis, and were not able to include an investigation of the suggested interaction between *ERBB2* and *TP53*. Additionally, while we controlled for other genomic factors confounding the relationship between *TP53* somatic mutation and immune signature status, it is possible that unaccounted for confounding occurred. Also, our study design was cross‐sectional, and lacked the ability to evaluate a temporal relationship between *TP53* somatic mutation and immune‐rich status. The exclusion of tumors in TCGA with pathologist‐determined cellularity of less than 60% (http://cancergenome.nih.gov/cancersselected/biospeccriteria) is a concern for selection bias and possible underrepresentation of immune‐rich, low cellularity tumors. In comparing immune measures to cellularity, we found no association between degree of correlation with immune signature and tumor cellularity in TCGA (Kruskal–Wallis, *p*‐value .45), or in METABRIC when comparing tumor cellularity and lymphocytic invasion status (chi square *p*‐value .08).

Use of separate measures for tumor immune invasiveness represented a trade‐off. We chose not to apply CIBERSORT to array data because it was developed for RNA‐Seq. Secondly, we wanted to confirm the relationship between *TP53* and immune invasion using pathologist‐confirmed, non in silico methods. Thus we chose to use METABRIC‐based measure of lymphocytic invasion. However, the incongruity between leukocyte (TCGA data) and lymphocyte (METABRIC data) cell types may present a possible concern for bias. In order to check the similarity between CIBERSORT and lymphocyte‐only measure, we compared previous measures of effector T cell and natural killer cell (NK) cytolytic activity for the same TCGA data (Rooney et al., 2014). Using a Kruskal–Wallis test, we found TCGA immune‐rich status to be strongly associated with increased cytolytic activity (*p* < 2.2e−16, data not shown). In addition, CIBERSORT is well documented as being able to identify tissue samples which were positive for at least one cell type with ≥ 94% sensitivity and 95% specificity(Rooney et al., 2014).

In general, *TP53*‐mutated tumors are significantly more immunogenic and have higher mutational burden, and lower overall variant allele frequency (more clonal heterogeneity). The relationship between clonal heterogeneity of tumor cells with *TP53* somatic mutations across the bulk tumor sample and tumor immune status suggests that tumors with a more homogenous population of cancer cells, have less overall mutations, and are also more immune‐poor. This is further supported through the observed median decrease in total burden of somatic mutations per immune‐poor tumor. Previous research using variant allele measures for clonal heterogeneity in breast cancer has used an overall, genome‐wide score (Quigley et al., 2015). We are the first to examine measures of clonal heterogeneity for a single gene (*TP53*) as they relate to somatic mutation and tumor immune status. Our results suggest that while immune‐rich tumors have high frequency of specific codons, differences in immune infiltration status were not due to clonal heterogeneity for individual codons. Specifically for p.R175H, we found that the median variant allele frequency for *TP53* did not differ significantly by immune signature group. This suggests that the mechanism for immunogenicity is independent of tumor clonal heterogeneity and due an unknown biologic mechanism of the mutation.

## CONFLICT OF INTEREST

The authors declare no potential conflict of interest.
